# Random forest methodology for model-based recursive partitioning: the mobForest package for R

**DOI:** 10.1186/1471-2105-14-125

**Published:** 2013-04-11

**Authors:** Nikhil R Garge, Georgiy Bobashev, Barry Eggleston

**Affiliations:** 1 Health Sciences Division, Social, Statistical and Environmental Sciences, Research Triangle Institute, 3040 Cornwallis Road, Cox 342, Research Triangle Park, NC, 27709, USA

**Keywords:** Random forests, Model-based recursive partitioning, Ensemble, R

## Abstract

**Background:**

Recursive partitioning is a non-parametric modeling technique, widely used in regression and classification problems. Model-based recursive partitioning is used to identify groups of observations with similar values of parameters of the model of interest. The *mob()* function in the party package in R implements model-based recursive partitioning method. This method produces predictions based on single tree models. Predictions obtained through single tree models are very sensitive to small changes to the learning sample. We extend the model-based recursive partition method to produce predictions based on multiple tree models constructed on random samples achieved either through bootstrapping (random sampling with replacement) or subsampling (random sampling without replacement) on learning data.

**Results:**

Here we present an R package called “mobForest” that implements bagging and random forests methodology for model-based recursive partitioning. The mobForest package constructs large number of model-based trees and the predictions are aggregated across these trees resulting in more stable predictions. The package also includes functions for computing predictive accuracy estimates and plots, residuals plot, and variable importance plot.

**Conclusion:**

The mobForest package implements a random forest type approach for model-based recursive partitioning. The R package along with it source code is available at http://CRAN.R-project.org/package=mobForest.

## Background

Recursive partitioning is a non-parametric modeling technique, widely used in regression and classification problems. Recursive partitioning methods like Random Forests™ [1] are able to deal with large number of predictor variables even in the presence of complex interactions. “Classification and regression trees” (CART) [2] is one of the most commonly used recursive partitioning methods that can select from among a large number of variables that are most important in explaining the outcome variable. The basic idea of CART algorithm is to sequentially split the data to identify groups of observations with similar values of response variable. During each step, a number of bivariate association models are run using every suspected predictor variable, and the one that has the strongest association with the response variable is selected. Then the data is split into two or more subgroups based on the optimal cutpoint in the selected predictor [3]. Thus the selected predictor becomes a partitioning variable. For binary predictor the split is unambiguous, but for a continuous one the bets split is used and the strength of association is usually adjusted for multiple choices, Strobl et al. 2009 [3]. The subrgoups formed by such split are often called nodes or “leafs”. The partitioning of the data continues till a stopping condition is met such as a) nodes contain observations of only one class, b) no predictor variable shows strong association within a given node, c) number of observations within a node are less than the specified minimum threshold.

Model-based recursive partitioning [4] partitions the groups of observations with similar model trends (between another predictor variable and the response variable). This is different from partitioning that identifies groups of observations that show similar value of the response variable. For example, a linear regression could be used to model the efficacy of treatments considered in a study. However, the treatment effects as well as the intercept parameter of this model may be different for different subgroups of patients. In this example, the model of interest relates treatment and clinical response but the model parameters can be different for different subgroups. “Model-based recursive partitioning” partitions the feature space to identify subgroups of patients with similar treatment effects and predicts clinical response based on the estimated treatment effects within different subgroups. The mob() function [4] implemented in the “party” package in R [5] allows one to perform model-based recursive partitioning. This function takes the model of interest and partition variables (covariates specifying the feature space that are used as splitting variables in a model-based tree) as input arguments and returns a tree with fitted models in each terminal node.

Regardless of the choice of recursive partitioning method (model-based or CART), single tree models could be instable to small changes in learning data. In other words, a slight change in learning sample can produce substantially different tree structures thereby inducing high variability in predictions obtained across trees [3]. Therefore, ensemble methods like “bagging” [6] and “random forests” - random selection of features (sets of predictor variables) - are commonly exercised to build large number of tree models and aggregate predictions cross the diverse set of trees to obtain stablepredictions [6-9]. Both the methods, bagging and random forests, construct trees on random samples of learning data. Random sampling is achieved either through bootstrapping (random sampling with replacement) or subsampling (sampling without replacement). Bagging, involves fitting trees to each random sample while considering the complete set of predictor variables during the process of splitting a tree node. “Random forests” produces a more diverse set of trees because at each level of a tree, a random subset of predictor variables is considered from which one might be selected for splitting the node. This allows a tree model to incorporate useful but weaker predictors that otherwise would be dominated by stronger predictors [3].

The main objective of this paper is to introduce the mobForest R package which implements random forest for both bagging and random variable selection methodology for model-based recursive partitioning. The mobForest package is available from the Comprehensive R Archive Network (CRAN) at http://CRAN.R-project.org/package=mobForest. This package computes predictions on multiple model-based trees that are constructed through random forest methodology. Predictions are aggregated across trees to produce stable predictions. The package provides functions to compute predictive accuracy estimates on individual trees and the complete mobForest. Predictive performance is computed on out-of-bag (OOB) cases – cases not used in a tree building process [4]. The metrics implemented to compute predictive performance are “pseudo R^2^” and mean square error (MSE) for continuous outcome and “proportion of correctly classified” (PCC) for binary outcome. The pseudo R^2^ predictive accuracy metric is defined as the proportion of total variation in outcome explained by the tree model (or forest). Both metrics “pseudo R^2^” and PCC range between 0 and 1. The mobForest package computes variable importance scores and provides functions to draw variable importance and predictive performance plots. This package can use multiple cores/processors for parallel computation. The parallel package that supports parallel computing (in R) is utilized for building trees on multiple cores/processors simultaneously. The computation time is greatly reduced if the analysis is run on a multi-core machine.

## Implementation

### Overview of functions available in mobForest package

The mobForest package contains functions for constructing model-based trees incorporating random forest methodology, computing predictions, predictive accuracy estimates, residuals plot, and variable importance plot. The detailed description of all the implemented functions is provided in the manual posted at CRAN (http://CRAN.R-project.org/package=mobForest). Here we outline most important functions.

#### Tree modeling

The main function used to develop model-based trees incorporating random forest methodology is called mob_rf_tree(). The mob_rf_tree() is a modified version of mob() function, implemented in the party package in R. The source code in the mob() function was modified such that a random subset of partitioning variables is selected during the process of splitting a tree node.. From this subset, a variable associated with the highest “parameter instability” [4] is selected as a splitting variable.

#### Setting up forest controls

Before starting the analysis the users are recommended to specify the parameters that control forest growth. The parameters can be set using mobForest_control() function that returns an object of S4 class mobForestControl containing forest controls. The parameters include:

• ntree: Number of trees to be constructed in mobForest (default = 300)

• mtry = number of input variables randomly sampled as candidates at each node (default is one-third of the number of partitioning variables).

• replace = TRUE/FALSE. replace = TRUE performs bootstrapping. replace = FALSE (default) performs sampling without replacement.

• fraction: number of observations to draw without replacement (default is 0.632). This parameter is relevant only if replace = FALSE).

• mob.control: Object, implemented in party package, used to set up control parameters for building model-based trees. A few important parameters in this object include:

○ alpha: A node is considered for splitting if the p value for any partitioning variable in that node falls below alpha (default 1).

○ Bonferroni: logical. Should p values be Bonferroni corrected? (default FALSE).

○ minsplit: integer. The minimum number of observations (sum of the weights) in a node (default 20).

### The main function mobForestAnalysis()

The mobForest package provides one main function called mobForestAnalysis() that takes all the necessary parameters as input arguments to start the mobForest analysis for model-based recursive partitioning. The input arguments to this function are kept similar to those in mob() from the party package, so users familiar with that function have an easy transition to using mobForestAnalysis(). mobForestAnalysis() takes following input parameters:

• formula: an object of class formula specifying the model that will be fit within each node. This should be of type y ~ x1 + … + xk where the variables x1, *x*2, …, xk are predictor variables and y represents an outcome variable. In this paper, this model will be referred to as the node model.

• PartitionVariables: A character vector specifying the partition variables used to build trees within the mobForest.

• Data: input dataset that is used for constructing trees in mobForest. Learning samples and out-of-bag (OOB) samples are created from this data (using bootstrapping or subsampling). The mobForest is constructed using learning samples and validated on out-of-bag samples.

• mobForest.controls: object of class mobForestControl returned by mobForest_control(), that contains parameters controlling the construction of mobForests.

• model: model of class StatModel used for fitting observations in current node, and it is used in the same manner as used in mob(). This parameter allows fitting a linear model or generalized linear model with formula y ~ x1 + … + xk. The parameter “linearModel” fits linear model. The parameter “glinearModel” fits Poisson or logistic regression model depending upon the specification of parameter “family” (explained next). If “family” is specified as binomial() then logistic regression is performed. If the “family” is specified as poisson() then Poisson regression is performed.

• family: a description of error distribution and link function to be used in the model, and it is used in the same manner as used in mob(). This parameter needs to be specified if generalized linear model is considered. The parameter “binomial()” is to be specified when logistic regression is considered and “poisson()” when Poisson regression is considered. The values allowed for this parameter are binomial() and poisson().

• newTestData: A data frame representing test data for independent validation of mobForest model. This data is not used in the tree building process.

• processors: the number of processors/cores on your computer. By default only one core is used for computations. If a computer has more than one core then increasing this variable to a value less than or equal to the number of cores will allow the package to exploit the multi-core parallelism and produce results relatively faster.

The function mobForestAnalysis() returns an object of class mobForestOutput which stores results from random forest analysis. This object stores predicted values, predictive accuracy estimates, residuals and variable importance scores produced during the analysis. This object is passed as an input argument to other functions to extract the relevant results.

#### Predictions

After constructing a model-based tree on a learning set using the function mob_rf_tree(), the predicted values for each subject are computed using the function treePredictions(). This function, called within mobForestAnalysis(), takes a dataset and a tree model as input arguments and returns fitted values of response variable on each observation within the dataset. Based on the characteristics of each observation, the treePredictions() function traverses through the tree model to an appropriate terminal node and obtains model parameters to compute fitted values of response variable. If the model of interest is logistic regression, then the fitted values are predicted probabilities of a classification (in one category). The mobForest package summarizes predictions obtained across multiple model-based trees. The Fitted values are averaged across the tree models (for each subject) and can be obtained using the function getPredictedValues(), which is a S4 method of class mobForestOutput. This function returns fitted values averaged on OOB data only, complete data or a new test data (supplied as a newTestData argument in the function mobForestAnalaysis()). The getPredictedValues() function takes three input arguments.

• mobForestOutput object - returned by mobForestAnalysis()

• OOB = TRUE/FALSE: OOB = TRUE (default) returns predictions across tree model on out-of-bag data (combined across all trees). OOB = FALSE returns predictions on complete data.

• Newdata = TRUE/FALSE. If newdata = TRUE, the OOB parameter is ignored and the predictions on the new test data, supplied as a newTestData argument to mobForestAnalysis(), are returned. newdata = FALSE (default) ignores newdata parameter and returns predictions based on the OOB parameter.

The function getPredictedValues() returns a matrix with 3 columns. The first column contains average predicted value for each subject across all the trees models. The predictions are averaged on OOB data, complete data or a new test data (as per the input parameter specification). The second column contains standard deviation of predictions, for each subject, across all the tree models. The third column contains residuals – difference between observed outcome and expected prediction - for each subject across the tree models. The residuals are reported only when linear or Poisson regression is considered as the node model.

### Predictive accuracy and error estimates

#### Metrics

The OOB cases provide a fair means of performance/error estimation based on training data alone. The predictive accuracy estimates are computed differently for a logistic regression model than linear or Poisson regression model. When linear or Poisson regression model is considered, predictive accuracy metric R^2^_k_ is defined as the proportion of total variation in outcome variable explained by the kth tree on OOB cases. In case of logistic regression model, the predicted probabilities for OOB cases are converted into classes (yes/no, high/low, etc. as specified) based on the probability cutoff specified by the end user (default is 0.5 if not specified) and predictive accuracy PCC_k_ is defined as the proportion of OOB cases correctly classified by the kth tree model. Both metrics PCC_k_ and R^2^_k_ range between zero and 1. In case of linear regression model, R^2^_k_ is a function of “sum of squared errors” (SSE_k_) and “total sum of squares” (SSTO_k_) on OOB data used to build the kth tree. It is computed as

(1) $$R_{k}^{2}=1-\frac{\mathrm{SS}E_{k}}{\mathrm{SST}O_{k}}$$

where,

(2) $$\mathrm{SS}E_{k}={\sum_{x=1}^{n}\left(y_{x}-{\hat{y}}_{x}\right)}^{2}$$

(3) $$\mathrm{SST}O_{k}=\sum_{x=1}^{n}{\left(y_{x}-\bar{y}\right)}^{2}$$

and y_x_ is the observed outcome for xth OOB case, ${\hat{y}}_{x}$ is the predicted outcome for xth OOB case, n is the number of OOB cases not considered in building kth tree, and $\bar{y}$ is the mean observed outcome of OOB cases. It should be noted that R^2^_k_ can be negative when SSE_k_ is greater than SSTO_k_. In such situations, we force R^2^_k_ to zero. The other metric used for measuring predictive accuracy is “mean square error” (MSE) [10]. MSE_k_ defined as the MSE estimate on OOB cases for the kth tree model and is calculated as follows.

(4) $$\mathrm{MS}E_{k}=\frac{\sum_{x=1}^{n}{\left(y_{i}-{\hat{y}}_{x}\right)}^{2}}{n}$$

Predictive performance is also estimated at “forest level” - after aggregating OOB predictions across all the trees and then computing R^2^ and MSE.

#### Predictive accuracy function

The function PredictiveAccuracy() (S4 method of class mobForestOutput) can be used to extract predictive accuracy estimates over OOB cases and/or a new test data. It takes three input arguments:

• mobForestOutput object

• Newdata = TRUE/FALSE. If newdata = TRUE, R^2^ (or PCC) and MSE are obtained for the new test data supplied as a newTestData argument to mobForestAnalysis(). newdata = FALSE (default) ignores newdata parameter and returns R^2^ (or PCC) and MSE estimates based on OOB predictions and complete dataset predictions summarized across all trees.

• plot = TRUE (default). This allows user to purview the distribution of R^2^ (or PCC) and MSE estimates for OOB cases across all the trees, overall R^2^ (or PCC) and MSE estimates when OOB predictions are aggregated across all the trees, and overall R^2^ (or PCC) and MSE estimates when predictions on new test data are aggregated across all the trees. plot = FALSE produces no plot.

PredictiveAccuracy() returns a list containing: a) OOB R^2^ (or PCC) estimates across all the trees, b) MSE estimates on OOB data across all the trees, c) overall R^2^ (or PCC) estimate when OOB predictions are aggregated across all trees, d) overall MSE estimate when OOB predictions are aggregated across all trees, e) R^2^ (or PCC) estimates on complete data across all the trees, f) MSE estimates on complete data across all the trees, g) overall R^2^ (or PCC) estimate when complete-data predictions are aggregated across all the trees, h) overall MSE estimate when complete-data predictions are aggregated across all the trees, i)the node model and partition variables used, j)if newdata = TRUE, overall R^2^ (or PCC) and MSE estimates when predictions on new test data are aggregated across all the trees.

**Variable importance** Variable importance assessment is a process of ranking variables in the predictor set according to their importance in producing accurate predictions. “Permutation accuracy importance” method [1,3,10] is used to compute importance scores for each variable. To determine the importance of a variable m, values of m in the OOB cases are randomly permuted and PCCp (proportion of OOB cases correctly classified when binary outcome is considered) or MSE_p_ (for continuous outcome) is obtained through variable-m-permuted OOB data. Next, MSE_p_ is subtracted from MSE_k_ (or PCCp is subtracted from PCCk) which was obtained using original un-permuted OOB data. The average of this number over all the trees in the forest is the raw importance score for variable m. One can invoke functions getVarimp() and varimplot() (S4 methods of class mobForestOutput) to produce variable importance scores and variable importance plot.

**Residual plot** One can invoke the function residualPlot() (S4 method of class mobForestOutput) to produce the following diagnostic plots.

• residuals vs. predicted outcomes for OOB cases: this plot should produce a distribution of points randomly scattered across 0, regardless of the size of the fitted value.

• histogram of OOB residuals: this plot is expected to be roughly normal with mean 0.

It should be noted that the above diagnostic plots are typical when the fitted values are obtained through linear regression but not when logistic or Poisson regression is considered as a node model. Therefore, mobForest package produces the above residual plots only when linear regression is considered. For logistic or Poisson models, a message is printed saying “Residual Plot not produced when logistic of Poisson regression is considered as the node model”.

## Results and discussion

We illustrate the use of mobForest package on alcohol treatment data from the COMBINE study [11]. The purpose of this study was to evaluate the efficacy of pharmaceutical and behavioral therapies (as well as combinations of the two therapy types) for treatment of alcohol dependence. The study enrolled 1383 alcohol-dependent adults (not experiencing significant alcohol withdrawal) from 11 different sites. Subjects were randomized into 8 treatment groups. There are 8 groups made up of 2 × 2 × 2 factorial design in treatments; naltrexone/placebo, acamprosate/placebo, and CBI + medical management/medical management only. The treatment duration was 4 months. The goal for this analysis was to estimate the effects of treatments on a continuous outcome variable “fifty.reduce” – indicator variable (0/1) representing if a subject reduced his/her drinks per drinking day by at least 50% from the baseline. Our node model of interest was

(5) $$\begin{array}{ll} \text{Log}\left(\frac{P\left(Y=1\right)}{1-P\left(Y=1\right)}\right) & =\beta_{0}+\beta_{1}X+\beta_{2}T_{2}\\ & \;+\beta_{3}T_{3}+\beta_{4}T_{4}+\beta_{5}T_{5}\\ & \;+\beta_{6}T_{6}+\beta_{7}T_{7}+\beta_{8}T_{8}\\ & \;+e \end{array}$$

where *Y* represents “fifty.reduce”, *X* represents baseline percent drinking days (bpdrkday), T_j_ is a 0/1 dummy variable representing the jth treatment,, *β*_*0*_ represents the intercept term of regression model, *β*_*1*_ represents baseline effect, *β*_*2*_,…,*β*_*8*_ represent the treatment effects for treatments *T*_*2*_,…, *T*_*8*_ (with treatment group 1 as a reference category), and *e* represents residuals . We used mobForest package to estimate the treatment effects for different groups of patients, partitioned through model-based recursive trees, and summarize outcome predictions across large number of trees.

**Figure 1:**
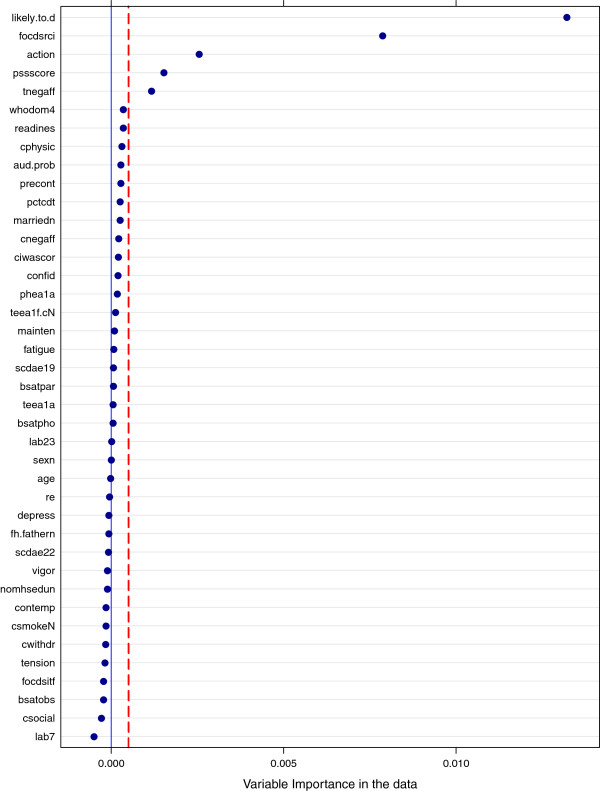
Variable importance plot for alcohol dependence study.

**Figure 2:**
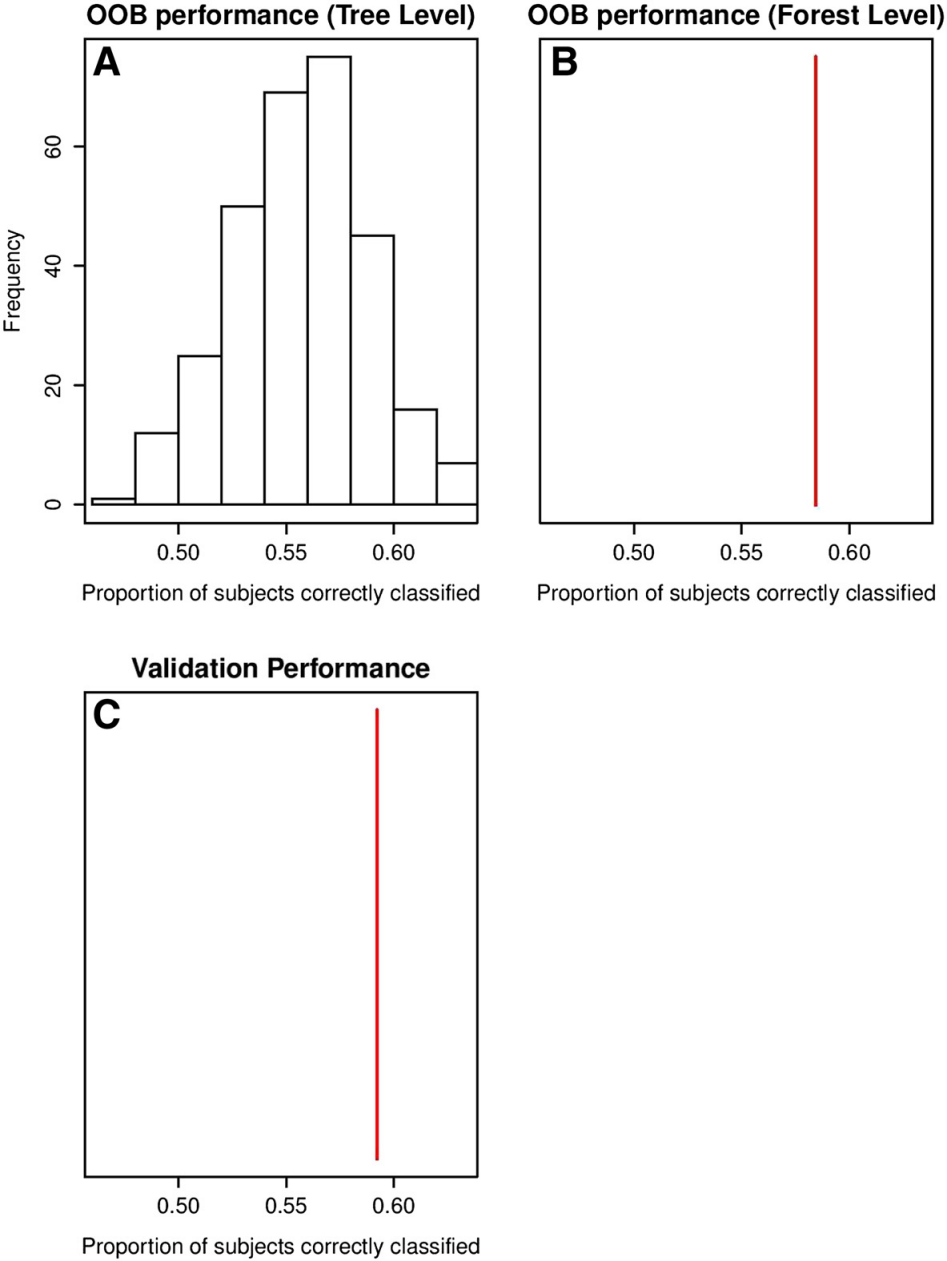
**Predictive Accuracy plots on alcohol data.** Figure **A** shows the distribution of PCC on OOB cases across all 300 trees. Figure **B** shows overall PCC estimate when the OOB predictions are combined across all trees. Figure **C** shows overall PCC estimate on the independent validation dataset.

Prior to the analysis, the data was loaded in R and partitioned into learning and validation sets. The training set contained 987 subjects (80%) and the independent validation set contained 233 subjects (20%). The validation set was used as an independent dataset for evaluating the performance of random forest model. We ran mobForestAnalysis() function on the training data with following forest settings: trees = 300, replace = F (for sampling without replacement), alpha = 0.5, bonferroni = T, and minsplit = 40 (minimum 40 cases in each terminal node). The parameter “prob.cutoff” was set to 0.5. Predicted probabilities, P(Y = 1), are converted to classes (0/1) based on the threshold “prob.cutoff” 0.5. A list of 40 variables was supplied as partition variables (used in splitting the nodes in trees). It took 28 minutes to perform mobForest analysis on a 32 bit machine with Microsoft windows XP Professional operating system. The machine had 4 gigabytes of RAM and Intel i7-2600 @ 3.40GHz CPU (with 8 cores). All the eight cores were used during the mobForest analysis on the COMBINE dataset.. The results (variable importance scores, predictions P(Y = 1) on OOB data, complete data, test data) were produced through this analysis. After producing results, we used the function varimplot() to produce a variable importance plot. The Figure 1 shows the variable importance plot. According to Figure 1, the top 5 variables show the strongest association with the treatment outcome include “likely.to.d” – likely to drink score, “focdsrci” – obsessive compulsive drinking scale, “action” - University of Rhode Island Change assessment score, “pssscore” – perceived stress score, and “tnegaff” – negative affect from alcohol abstinence on self efficacy. Then we produced plots of predictive accuracy estimated across individual trees using the function PredictiveAccuracy(). Figure 2 shows the predictive performances (PCC – proportion of correctly classified) on OOB data and the independent validation set. Figure 2 also reports the predictive accuracy estimates at “forest level” - after aggregating predictions (P(Y = 1)) of subject across all the trees. This plot is not a comparison of performance of single trees to the performance of a forest, but simply a graphical representation of selected measures of predictive accuracy. The overall PCC estimate measured after combining OOB predictions across all 300 trees is 0.58 (577 out of 987 cases were correctly classified at “prob.cutoff” 0.5). The PCC estimate on the independent validation dataset was 0.59. The “tree level” performance estimates obtained on OOB cases ranged from 0.46 - 0.63. We also computed area under ROC curve (AUC) for predictions obtained on training and independent validation datasets. AUC was computed using Wilcoxon-Mann–Whitney statistic implemented in “ROCR” package in R. AUC estimate on training dataset was 0.81 and validation dataset was 0.66.

We also did fit the learning data using a logistic regression model containing all the parameters in equation (5) plus the best subset of partition variables - selected through stepwise regression analysis with forward selection procedure. Four of the top 5 important variables obtained through mobForest analysis were also selected in the final model obtained using stepwise regression. These variables include “likely.to.d”, “focdsrci”, “action”, and “pssscore”. The AUC estimate for predictions obtained through stepwise regression on the training dataset was 0.71 and validation dataset was 0.60. Therefore, mobForest showed better performance than the stepwise regression method.

## Conclusions

The R package mobForest implements random forest method for model-based recursive partitioning. This package combines predictions obtained across diverse set of trees to produce stable predictions. The mobForest provides functions for producing predictive performance plots, variable importance plots and residual plots using data contained in the mobForest object. The package uses multiple cores/processors to perform parallel computations. The parallel package that supports parallel computing (in R) is utilized for faster computation. The mobForest package supports linear, Poisson and logistic regression models for use in model-based random forest type analysis.

## Availability and requirements

**Project name:** Alcohol dependence study

Project Home page:

**Operating system:** windows platform

**Programming Language:** R

**License:** GPL (≥2)

## Competing interest

The authors declare that they have no competing interest.

## Authors’ contributions

BE conceived the project and oversaw its design. NRG and BE developed the algorithm. NRG packaged the classes and functions into an R package and drafted the manuscript. BE and GB contributed in writing sections of the manuscript. All the authors read and approved the final manuscript.
